# Acute Pancreatitis as a Complication of Intragastric Balloon

**DOI:** 10.7759/cureus.16710

**Published:** 2021-07-29

**Authors:** Hussain A Al Ghadeer, Bashayer F AlFuraikh, Ahmed M AlMusalmi, Lamis F AlJamaan, Ezzeddin Kurdi

**Affiliations:** 1 Paediatrics, Maternity and Children Hospital, AlAhsa, SAU; 2 Internal Medicine, King Faisal University, AlAhsa, SAU; 3 Internal Medicine, King Fahad Hospital Hofuf, AlAhsa, SAU; 4 Gastroenterology, King Fahad Hospital Hofuf, AlAhsa, SAU

**Keywords:** acute pancreatitis, intragastric balloon, bariatric surgery, obesity., balloon pancreatitis

## Abstract

The intragastric balloon is a common minimally invasive procedure used prior to bariatric surgery for weight reduction. There are complications of this balloon with varying degrees of severity ranging from mild to severe life-threatening complications. Acute pancreatitis due to direct compression or catheter migration of the balloon should be considered in these patients. In the literature, there is little evidence that intragastric balloons could cause acute pancreatitis. We present two cases in which they had a history of IGB insertion complicated by acute pancreatitis. The diagnosis of acute pancreatitis due to the intragastric balloon was made after excluding other possible causes of acute pancreatitis. Both patients were hospitalized and managed conservatively.

## Introduction

Obesity is considered an epidemic disease, a serious public health issue, and is associated with increased morbidity, mortality, and decreased quality of life. Obesity has increased in recent decades, more than 1.4 billion adults worldwide are overweight or obese, and it is a leading public health concern globally [1]. There are different methods for treating obesity, such as invasive surgical methods as bariatric surgery and non-surgical as lifestyle modification and non-invasive procedures methods like intragastric balloons (IGBs). All of these methods have their indications with variable degrees of efficacy and safety [2]. Bariatric surgery is the most effective management option for those with morbid obesity with the desire to improve weight loss and reduce or treat obesity-caused comorbidities [3]. One of the minimally invasive bariatric procedures used is endoscopic IGBs; it is a type of restrictive therapy and has good efficacy, low cost, and low morbidity and mortality [4,5]. However, IGB insertion causes diverse complications ranging from mild complications such as nausea, abdominal pain, and gastroesophageal reflux to severe life-threatening ones, including ulceration, perforation, and bowel obstruction [1].

## Case presentation

Patient A

This 27-year-old Saudi male was not known to have any medical illness; he presented to the emergency department with a history of epigastric pain that started two days before admission. The pain was continuous, started suddenly with severity of 10/10 according to the patient with no diurnal variation or radiation. Moreover, it was sharp in nature, aggravated by movement and relieved by rest. The pain was associated with nausea and vomiting (gastric content). There was no history of change in bowel habits, urine, nor cardiopulmonary symptoms. The patient consumes alcohol on rare occasions, approximately one to two cups per month, and is known to be a smoker. The patient denied any history of abdominal trauma, scorpion bites, current medication use, or previous similar complaint. Family history was insignificant of autoimmune disease, inherited diseases, or similar complaints. The patient's surgical history was unremarkable. The patient was known to have morbid obesity, for which he underwent a minimally invasive procedure; an IGB was inserted seven months ago. As a result, he lost 17 kg, from 98 kg to 81 kg.

On examination, the patient was hemodynamically and vitally stable. The abdominal examination revealed severe epigastric tenderness, with typical bowel sounds and soft lax consistency in other regions. Systemic examination was unremarkable.

**Figure 1:**
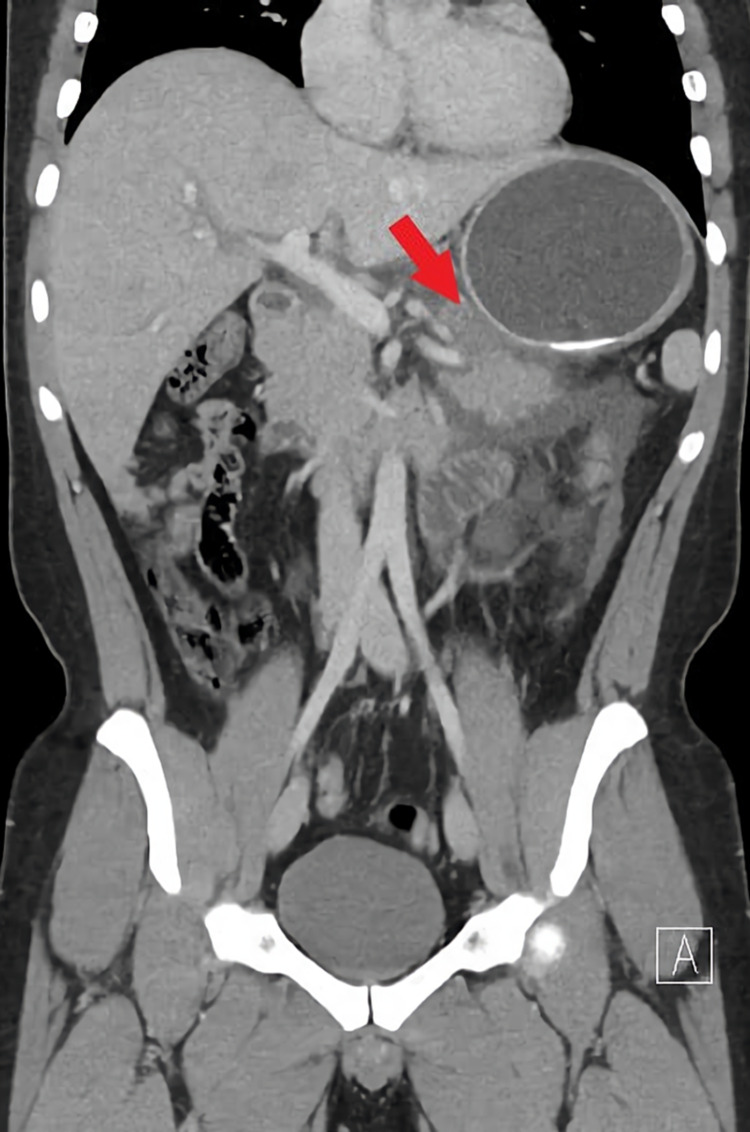
Necrotizing pancreatitis as a mass effect of the balloon on the pancreas

On investigations, laboratory, complete blood count, renal and liver function tests were within normal limits. Amylase and lipase were above the average level with the values of 327 and 2,000, respectively (Table 1). Imaging showed ultrasound was nil for gallbladder stones and common bile duct dilatation. CT scan of the abdomen showed necrotizing pancreatitis with compression of the pancreas by IGB (Figure 1).

**Table 1:** Laboratory and imaging investigations

Laboratory investigations	Patient A	Patient B	Reference level
Complete Blood Count			
White Blood Cells (WBCs)	10.43	18.16	10^9/L (4-10)
Red Blood Cells (RBCs)	5.65	4.72	10^12/L (3.8-4.8)
Hemoglobulin	16.50	13.70	12-15 g/dL
Platelets	274	287	10^9/L (130-400)
Renal Profile			
Urea	4.30	1.80	1.7-8.3 mmol/L
Creatinine	71	54	53-120 µmol/L
Calcium Total	2.23	2.07	2.1-2.6 mmol/L
Sodium Serum	135	139	133-148 mmol/L
Potassium Serum	4.13	3.30	3.4-5.1 mmol/L
Chloride Serum	101.40	105	98-107 mmol/L
Liver Profile			
Aspartate Aminotransferase	23	28	0-40 U/L
Alanine Aminotransferase	38	67	30-65 U/L
Alkaline Phosphate	83	99	50-136 U/L
Total Bilirubin	9.40	7.10	0-24 µmol/L
Direct Bilirubin	2	1.22	0-5 µmol/L
Lipid Profile			
Cholesterol	4.15		<5.2 mmol/L
Triglyceride	0.70		0.45-1.81 mmol/L
High-Density Lipoprotein	1.18		0.8-1.8 mmol/L
Low-Density Lipoprotein	2.65		2-4 mmol/L
Inflammatory Marker			
C-reactive Protein	3.35 mg/dL		0-0.8 mg/dL
Pancreatic Enzyme			
Amylase	327	291	20-115 U/L
Lipase	2,000		10-140 U/L
Imaging			
Ultrasound	No abnormality detected	No abnormality detected	
CT Scan	Evidence of intragastric balloon. There is an area of enlargement and low attenuation/hypoenhancing in all phases involving the body of the pancreas and peripancreatic free fluid. Suggestive features of necrotizing pancreatitis.	The pancreas is mildly edematous with homogeneous enhancement with surrounding mild fat stranding and trace of free fluid suggestive of acute interstitial edematous pancreatitis. No evidence of collection. No evidence of CBD dilatation, biliary stone or dilated biliary radicles.	

The patient was treated conservatively under nothing per oral, intravenous fluids, antiemetics, and analgesia. The patient improved clinically and was discharged after three days of hospitalization without the removal of IGB.

Patient B

This 44-year-old Saudi female was not known to have any medical illness, presented to the emergency department with a history of epigastric pain three days earlier to admission. The pain was continuous, started suddenly, and was 9/10 in severity according to the patient with no diurnal variation. Pain radiated to the back, stretching in nature, aggravated by movement, and relieved by rest. The pain was associated with nausea and vomiting (gastric content). There was no history of change in bowel habits, urine, or cardiopulmonary symptoms. The patient was neither a non-smoker nor an alcoholic. The patient denied any history of abdominal trauma, scorpion bites, recent medication use, or previous similar complain. Family history was insignificant for autoimmune disease, inherited diseases, or similar complaints. The surgical history was clear. The patient was known to have morbid obesity, for which she underwent a minimally invasive procedure; an IGB was inserted three weeks ago. She lost 20 kg, from 130 kg to 110 kg. Eight months ago, the patient was COVID-19 positive, for which she was managed in the intensive care unit for three days.

On examination, the patient was hemodynamically and vitally stable. The abdominal examination revealed severe epigastric tenderness, normal bowel sounds, and soft lax consistency in other regions. No abnormalities were detected on systemic examination.

**Figure 2:**
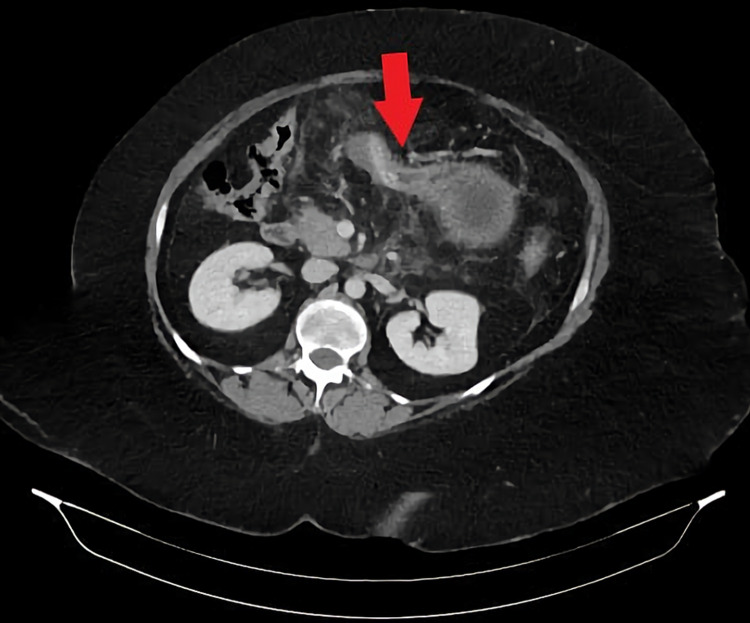
Mass effect of the balloon on the pancreas and dislodgment of the catheter into the duodenum

On investigation, laboratory, complete blood count showed leucocytosis, renal and liver function tests were within normal limits. Amylase was above the average level values 291 (see Table 1). Imaging showed ultrasound was nil for gallbladder stones or common bile duct dilatation. CT scan of the abdomen showed acute interstitial edematous pancreatitis with compression of the pancreas by balloon. Furthermore, there was dislodgment of the catheter into the duodenum (Figure 2).

The patient was treated conservatively by nothing per oral, intravenous fluids, antiemetics, and analgesia. The patient improved clinically and was discharged after three days of hospitalization without the removal of IGB. However, after two weeks of discharge, the patient presented with a similar picture in a more severe form, indicating the removal of IGB for improvement.

## Discussion

Obesity is a major health issue worldwide, with an estimated prevalence of 39% of the adult population being overweight, and 13% being obese [6]. In the Middle East, countries carry a burden of increasing rates of obesity and associated non-communicable diseases (NCDs). For example, the Kingdom of Saudi Arabia (KSA) ranks among the top countries of obesity with an estimated prevalence of about 67.5% of males and 69.2% of females are overweight. Furthermore, around 29.5% of males and 39.5% of females are obese [7]. Obesity is a well-known predisposing factor for comorbidities such as hypertension, cardiovascular diseases, diabetes mellitus type II, and cancer. There are different methods for treating obesity ranging from lifestyle modification to bariatric surgery - these methods show a varying degree of efficacy and safety. The IGB is commonly used as a minimally invasive procedure, and it is a relatively safe option to achieve short-term weight loss with satisfactory outcomes. It works as mechanical gastric distension, leading to the feeling of satiety rapidly, which results in decreased food intake [2]. IGB is inserted by endoscopy and filled with 400-700 mL of fluid. It is used temporarily up to six months prior to bariatric surgery then removed to achieve approximately 15%-20% of total body weight loss [8]. There are certain complications of IGB with varying degrees of severity ranging from mild symptoms including nausea, vomiting, abdominal pain, and gastroesophageal reflux to life-threatening complications including ulceration, perforation, and balloon migration [2]. Rarely, acute pancreatitis (AP) may also occur as a serious complication, as presented in this case. In this case, both patients A and B, medically free, underwent IGB that was filled with 606 mL and 678 mL of fluid, respectively. Patient A lost 17% of his total body weight after insertion of IGB for a seven-month period. Although it is unexpected for AP to present after seven months of IGB insertion, similar literature reported balloon-induced AP after eight months of IGB insertion, managed by IGB removal [9]. Other possibilities that might have triggered AP development, such as concurrent heavy meal intake that might have increased pancreatic compression, should be considered as well. Furthermore, the history of alcoholism, in this case, may raise the suspicion of alcoholic pancreatitis. Although the patient consumes 1-2 drinks per month, many studies report that 4-7 drinks per day (50-80 g) are usually required to cause AP. Nevertheless, it could be found in some individuals with low intake as low as 20g per day [10]; both are unlikely in our case. Patient B lost 15% of her total body weight after insertion of IGB three weeks earlier. Although IGB is commonly used and most adverse complications have been reported, other complications are not yet recognized. One of the rare adverse events of IGB is AP. There are a few reported cases in the literature of AP induced by IGB. AP is an inflammatory syndrome of the pancreatic gland initiated by an acute injury [11]. Various causes were identified in which gallstones and excessive alcohol consumption are the most common etiologies [12]. Severity may range from mild and self-limiting to extremely severe pancreatic necrosis and hemorrhage [13]. Diagnosis of AP is made with two out of three criteria: (1) Acute onset upper abdominal pain, (2) increase in serum amylase or lipase level by at least three times the upper limit of the normal range, and (3) characteristic findings on cross-sectional imaging (contrast CT, MRI, or ultrasound) [14]. IGB-related pancreatitis or balloon pancreatitis is thought to be due to the mass effect of the balloon on the pancreas or catheter dislodgment in the second part of the duodenum [15]. The patient typically presents with clinical manifestations similar to other AP causes, including severe epigastric pain radiating to the back and relieved by leaning forward, associated with nausea, vomiting, and fever. Moreover, laboratory investigations of AP usually reveal leucocytosis in CBC with a high level of inflammatory marker C-reactive protein, and most importantly, an increase in the levels of serum amylase and lipase [16]. In this current case, both patients A and B presented with sudden severe epigastric pain associated with nausea and vomiting with no change in bowel habits or history of trauma or chronic diseases. Laboratory revealed typical CBC values, renal and liver profiles. Serum amylase and lipase of both patients A and B were three times above the normal range. Imaging investigation: contrast CT scan of the abdomen is the modality of choice for assessing and diagnosing balloon pancreatitis. It usually shows the presence of IGB filling the stomach with features of acute interstitial pancreatitis. Features of pancreatitis include diffuse enlargement with peripancreatic inflammation and fat stranding. Furthermore, evidence of inflated balloon causing mechanical pressure on the pancreatic parenchyma and obstructing the pancreatic duct [16]. In our case, the CT scan of patient A showed necrotizing pancreatitis with compression of the pancreas by IGB. On the other hand, the CT scan of patient B showed interstitial edematous pancreatitis features and the possibility of the ampulla of Vater obstruction secondary to IGB catheter migration. There were two case reports where AP was developed due to IGB dislodgment to the duodenum; removing the IGB treated both cases. Consequently, rapid clinical and laboratory improvement was observed [17,18]. CT scan is also used to exclude other differential diagnoses, early detection of complications of pancreatitis, or complications related to IGB, including gastric outlet obstruction, gastric ulceration, perforation, and balloon migration. In addition, ultrasound is used to assess gallbladder diseases as a cause of AP. The definitive treatment of balloon pancreatitis is the removal of IGB, which leads to significant resolution of symptoms and normalization of abnormal laboratory and imaging investigations [16]. In this current literature, both cases were diagnosed as AP based on the clinical manifestation, laboratory, and radiological evidence of AP due to IGB. Both patients were managed conservatively, except patient B's IGB was removed after relapse of symptoms.

## Conclusions

AP is an unrecognized complication of IGB insertion. It presents a triad of history of a recent gastric balloon insertion, symptoms consistent with AP, and radiological and biochemical evidence of pancreatitis. Therefore, the correlation of clinical presentation and radiological findings is crucial in achieving an accurate diagnosis. Further studies are needed to determine the impact of balloon size and weight changes in the occurrence of AP. In addition, long-term outcomes of conservative management and determination of balloon removal as definitive management should be investigated.
